# Effect of Two Different Intraoral Polishing Systems on Surface Roughness, Color Stability, and Bacterial Accumulation of Zirconia-Reinforced Lithium Silicate Ceramic

**DOI:** 10.1055/s-0044-1779423

**Published:** 2024-03-31

**Authors:** Fatma Makkeyah, Dina Mohamed Moustafa, Mahmoud M. Bakr, Mahmoud Al Ankily

**Affiliations:** 1 Department of Fixed Prosthodontics, Faculty of Dentistry, The British University in Egypt, Cairo, Egypt; 2 Department of Medical Science, Faculty of Dentistry, The British University in Egypt, Cairo, Egypt; 3 General Dental Practice, School of Medicine and Dentistry, Griffith University, Gold Coast, Queensland, Australia; 4 Department of Oral Biology, Faculty of Dentistry, The British University in Egypt, Cairo, Egypt

**Keywords:** ceramic, color stability, polishing systems, zirconia

## Abstract

**Objective** The aim of this study was to investigate the effects of two intraoral polishing methods on zirconia-reinforced lithium silicate ceramic after ultrasonic scaling.

**Materials and Methods** Thirty disc-shaped samples of zirconia-reinforced lithium silicate were constructed. Freshly extracted bovine teeth were collected and cleaned then the discs were cemented into a cavity prepared onto their labial surface. The samples were divided into three groups (10 samples per group); S: Scaling only, SE: Scaling followed by polishing using Eve Diapro lithium disilicate polishers, SD: Scaling followed by polishing using Diatech ShapeGuard ceramic polishing plus kit. The surface roughness was evaluated after scaling and polishing the samples. For color stability, the samples were stored for 12 days at 37°C in an incubator to simulate 1-year consumption of coffee. L*a*b* color parameters were assessed using VITA Easyshade Advance 4.0 before and after the staining procedure and the color difference was measured. Finally, bacterial accumulation was evaluated by incubating the samples with a suspension of *Streptococcus mutans* ( *S. mutans* ), after that the *S. mutans* colonies were counted to obtain the values of colony-forming units (CFU). The final overall roughness, change in color and bacterial count were compared between all groups using one-way ANOVA and Tukey's post-hoc analysis. The Pearson correlation coefficient was used to determine the correlation between continuous variables. The cutoff for significance was chosen at *p* ≤ 0.05.

**Results** Scaling induced surface roughness of the zirconia-reinforced lithium silicate ceramic was significantly decreased after using both intraoral polishing systems and this was accompanied by a significant decrease in color change and bacterial count.

**Conclusion** Intraoral polishing techniques can reduce the roughness of the surface of zirconia reinforced lithium silicate restorations induced due to scaling and subsequently reduce the stainability and bacterial accumulation.

## Introduction

A commonly used material for ceramic restorations is lithium disilicate (LS) glass-ceramic. It contains 70% of LS orthorhombic crystal phase (Li _2_ Si _2_ O) in an amorphous matrix which provides a superior esthetic appearance than high-strength polycrystalline alternatives. 1 Unfortunately, because of their lower mechanical qualities, their usage in the molar region is limited. 2 Zirconia-reinforced lithium silicate (ZLS) is a novel class of computer-aided design and computer-aided manufacturing (CAD/CAM)-fabricated materials that combines zirconia's advantageous mechanical properties with the esthetic appeal of glass-ceramic materials. It is composed of a lithium-metasilicate glass-ceramic (Li _2_ SiO _2_ ) which is reinforced with around 10% zirconium dioxide (ZrO _2_ ) that forms a microstructure with fine grains of Li _2_ O-ZrO _2_ -SiO _2_ after the crystallization process. The material could be etched and cemented adhesively. 3

Previous studies demonstrated that dental plaque accumulation is considered a primary factor in periodontal disease as well as in dental caries. 4 5 6 To maintain oral health, regular oral examinations and intraoral scaling are therefore deemed necessary. 7 8 Calculus and dental plaque accumulated on the dental restorations and on surfaces of teeth are removed by intraoral scaling to provide low-energy clean surfaces. 9 The scaling procedure can be performed using either a periodontal curette or an ultrasonic scaler. However, using an ultrasonic scaler may result in increased roughness and alter the topography of the smooth surfaces of restorations, 10 11 which may influence the accumulation of microorganism colonies and dental plaque. 12 13 It is therefore essential to have a smooth surface on dental restorations for esthetics as well as for biological health, to enhance the durability and increase the strength of the restored tooth. 14 15

Intraoral polishing of restorative materials after scaling may be used to minimize the changes in surface roughness. 16 17 Many polishing protocols have been introduced to decrease the surface roughness and provide a smooth surface for ceramic restorations. These protocols include the use of diamond burs of different grit, polishing stones, sandpapers, abrasive rubber wheels, abrasive diamond particles, or diamond pastes. 14 16 18

Reviewing the literature, several studies investigating the influence of prophylactic periodontal scaling procedure on different restorative materials and the surface alterations and its relation to scaling methods have been found; however, few studies have evaluated the surface alterations caused by periodical ultrasonic scaling followed by intraoral polishing. 19 20 In the present study, the effect of repeated prophylactic periodontal scaling followed by polishing with various intraoral polishing systems on the roughness of the ceramic surface, color changes, and bacterial count in ZLS materials was evaluated. The null hypothesis of the current study was that there is no difference between different polishing kits and no difference between polished and unpolished groups.

## Materials and Methods

**Fig. 1:**
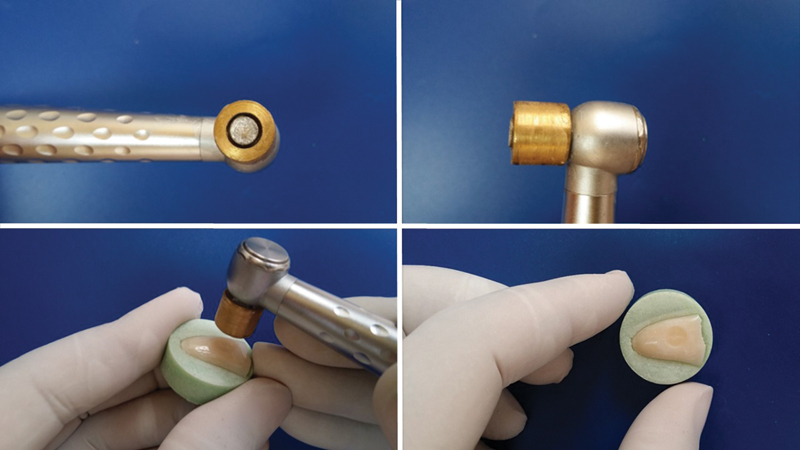
Steps of standardized cavity preparation.

The study experimental procedures were ethically reviewed by the The British University in Egypt Faculty of Dentistry Research Ethics Committee (Research Approval Number: 22-038). Thirty bovine anterior teeth were collected. All soft tissue was removed until teeth were visually clean. The teeth were decapitated 2 mm below the cemento-enamel junction using tapered diamond stone with copious coolant to remove the root. The labial surfaces of the teeth were flattened using a cylinder diamond stone to obtain a flat area of 1 × 1 cm ^2^ . The teeth were mounted in acrylic (Acrostone Dental Factory, Egypt) blocks using polyvinyl chloride tube of 1-inch inner diameter and 1.5 cm thickness. Then a cavity of 5 mm diameter and 0.5 mm depth was prepared on the flat surface of the bovine teeth using wheel diamond stone of 5 mm diameter (Komet Dental, Gebr. Brasseler). To standardize the depth of the cavity, a specially designed copper limiting tube and calibrating mold of 0.5 mm depth were constructed. The calibrating mold has a rounded cavity of 0.5 mm diameter and 0.5 mm depth. The limiting tube was cemented to the contra-angle handpiece using cyanoacrylate adhesive. The calibrating mold was then used to adjust the length of the projecting part of the wheel diamond stone. The cavity of the calibrating mold was pushed against the diamond stone until the calibrating mold touches the limiting tube leaving 0.5 mm of the diamond stone projecting from the limiting tube. With the wheel diamond stone and the limiting tube in place, cavities of 0.5 mm diameter and 0.5 mm depth were prepared on the flat labial surface of the teeth. The wheel diamond stone cuts the tooth structure until the depth of the cavity is 0.5 mm and it stops cutting when the limiting tube comes in contact with the flat labial surface ( Fig. 1 ).

Following the manufacturer's instructions, 30 disc-shaped samples of zirconia lithium silicate (VITA SUPRINITY PC, VITA Zahnfabrik) of 0.5 mm thickness and 5 mm diameter were constructed. Finally, the discs were finished, polished, and glazed with Vita Akzent plus Glaze paste on one surface of each disc.

The fitting surfaces of the ceramic discs were etched using hydrofluoric acid (9.5%) (BISCO, Inc, United States) porcelain etchant followed by porcelain primer (prehydrolyzed silane primer) (BISCO, Inc) application according to the manufacturer's instructions. The cavities on the teeth surfaces were acid-etched using phosphoric acid (37%) (Eco-Etch, Ivocalr Vivadent) followed by the bonding agent (TE-Econom Bond, Ivocalr Vivadent) according to the manufacturer's instructions. Finally, the discs were cemented into the cavities using Variolink N clear (Ivocalr Vivadent, Inc).

The samples were then randomly assigned to three groups (each of 10 samples); C: control (scaling only), SE: scaling followed by polishing using Eve Diapro LS polishers (diamond-impregnated 2 stages polishing system), SD: scaling followed by polishing using Diatech ShapeGuard ceramic polishing plus kit (diamond-impregnated 3-step silicone polishers).

**Fig. 2:**
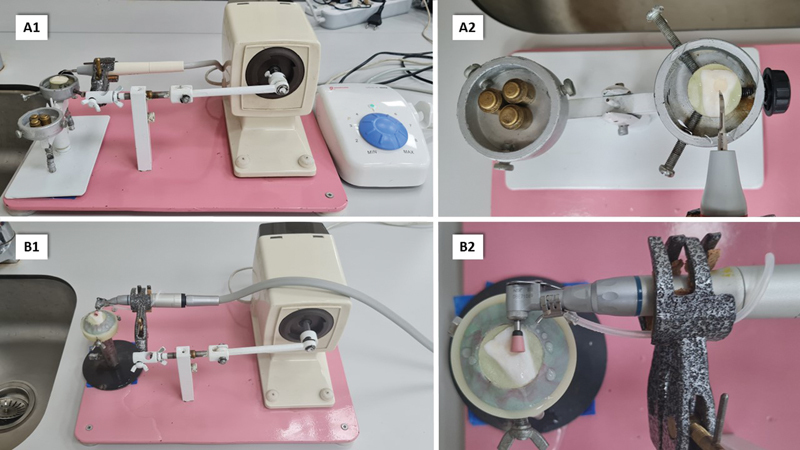
Scaling apparatus ( **A1** ) with sample holder and double pane balance ( **A2** ). Polishing apparatus ( **B1** ) with magnification of polishing procedure ( **B2** ).

For the standardization of the scaling and polishing procedures, a specially designed and constructed apparatus was used ( Fig. 2 ). The samples were stabilized by screws onto a pane of a double-pane balance. The scaling technique was performed at an intermediate power setting utilizing an ultrasonic scaler handpiece (Woodpecker Medical Instrument Co., Ltd) with the scaling tips at a right angle to the surface of the sample. A counterweighed balance was raised vertically, exerting a steady force of 30 *g* at the tip. A standard horizontal movement of 5 mm and three cycles of 20 seconds each were performed by the ultrasonic handpiece at a 2-Hz speed. 21 22 Scaling was performed on all groups, then group SE was polished using Eve Diapro LS polishers and group SD was polished using Diatech ShapeGuard ceramic polishing plus kit. The polishing process was performed following the manufacturer's recommendations. Using a low-speed handpiece, each instrument was utilized for 30 seconds in a single direction. Finally, air-water spray was used to rinse the samples for 15 seconds and then ultrasonically cleaned in 100% distilled water for 1 minute and then air dried.

A profilometer, Surface Roughness Tester TIME3202 (TR220) (Landmark Industrial Inc, United States), was used to assess the surface roughness of all samples. A cutoff of 0.25 mm, the number of cuts 1, and range ± 40 μm was used. To calculate the surface roughness (Ra) values, measurements were taken in three different regions for each sample and averaged to determine the mean values. A general purpose surface optical profiler, ZYGO Maxim-GP 200 profilometer, was used to assess the topography and microstructure of surfaces of the samples in three dimensions. It employs a computerized phase stepping interferometry upgraded with scanning white light interferometry and highly developed surface texture software that analyzes areas as well as profiles and step height. An interferometric objective (Michelson or Mirau type) is controlled by scanning white light fringes and receiving white light from a halogen lamp incident on an optical cube beam splitter. The interference fringes are created when the light that is reflected from the samples returns through the interferometric objective and travels through the cube beam splitter to the camera. Using a computer and advanced texture analysis software, all the surface-related data can be obtained.

*Staining procedure* : Coffee solution (Nescafé Classic; Nestlé Egypt) was used for the staining procedure.

All samples were stored in coffee solution for 12 days at 37°C, which is the equivalent of 1 year's worth of coffee intake, 23 24 in an incubator (Model B 28, BINDER GmbH). The manufacturer recommended using 3.6 *g* of coffee along with 300 mL of hot water. Filter paper was used to filter the solution after it had been agitated for 10 minutes (Melitta; Melitta Haushaltsprodukte GmbH & Co Kg). 23 Every 12 to 12 ± 1 hour, the solution was stirred. After 12 days, the samples were washed with tap water and dried with tissue paper.

Using VITA Easyshade Advance 4.01 (VITA shade, VITA made, VITA), color parameters for each sample were recorded before and after the staining process following the CIE L*a*b* color ordering system then the color difference was calculated according the following formula: Δ *E* = ([ *L*_2_ – *L*_1_ ] 2 + [ *a*_2_ – *a*_1_ ] 2 + [ *b*_2_ – *b*_1_ ] ^2^ ) ^1/2^ .

*Bacterial accumulation test* : A standard strain of *Streptococcus mutans* , ATCC 25175, was used to create a standard suspension of *S. mutans* . It was cultured on brain heart infusion agar media (Oxoid, Untied Kingdom), and it was then incubated for 24 hours at 37°C in a CO _2_ atmosphere. Colonies were placed in a spectrophotometer while suspended in sterile physiological saline containing 0.9% sodium chloride (NaCl) (PG Instruments Ltd, United Kingdom). To get a normal suspension with 106 cells/mL, the optical density (OD398) was changed to 0.620. These parameters were formerly established using a standard curve for colony-forming unit (CFU) versus absorbance.

A broth containing 20 g trypticase, 2 g NaCl, 3 g K _2_ HPO _4_ , 2 g K _2_ HPO _4_ , 1 g K _2_ CO _3_ , 120 mg MgSO _4_ , 15 mg MnSO _4_ , 50 g C _6_ H _8_ O _7_ , and 20 g sucrose, dissolved in 1,000 mL of distilled water, was used for the biofilm formation assay. The broth was autoclaved at 121°C for 15 minutes to sterilize it. Using sterile forceps, sterilized specimens were inserted into a sterile 24-well plate with 1.5 mL of medium. Each well received 150 mL of bacterial suspension to create biofilms. The plate was sealed and incubated for 24 hours at 37°C in a CO _2_ chamber. After that, samples were taken out and washed twice in sterile physiological saline to get rid of any loosely bound microbial cells. Then, to disperse biofilm, each specimen was put into a sterile falcon tube with 3 mL of brain heart infusion broth (Oxoid). The obtained suspensions were diluted five times in sterile physiological saline. Ten microliters of each suspension were spotted in duplicates in a CO _2_ incubator for 24 hours at 37°C on brain heart infusion agar plates. Mean CFU values were calculated based on the counting of plates containing 3 to 30 colonies. 25 26

Statistical Package for Special Science (IBM SPSS Statistics 26) was used to analyze the data. To judge the normality of the data, the Kolmogorov–Smirnov and Shapiro–Wilk tests were applied. Information was presented as mean and standard deviation (SD). The change in color, bacterial count, and final overall roughness was compared between all groups using one-way analysis of variance and Tukey's post-hoc analysis. The Pearson's correlation coefficient was used to determine the correlation between continuous variables. The cutoff for significance was chosen at *p* ≤ 0.05.

## Results

**Fig. 3:**
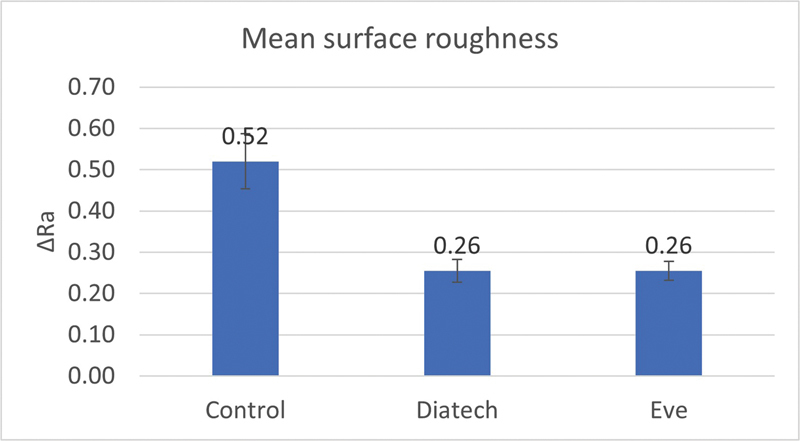
Bar chart representing mean values of surface roughness.

Mean values of total surface roughness for the control group (Ra = 0.52 ± 0.09 µm) were statistically significantly different from both polishing groups (SE: Ra = 0.26 ± 0.032 µm) and (SD: Ra = 0.26 ± 0.038 µm) ( *p* ≤ 0.001, degrees of freedom [df] = 2, 27, *F* = 62.423), while the two polishing groups were not statistically significantly different ( Fig. 3 ).

**Fig. 4:**
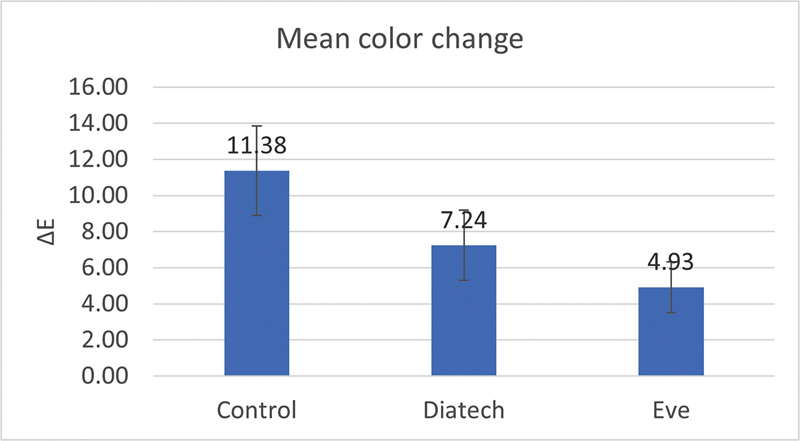
Bar chart representing mean values of color change.

Similarly, the color change of the control group (∆ *E* = 11.38 ± 3.46) was statistically significantly different from both polishing groups (SE: ∆ *E* = 4.93 ± 1.97) and (SD: ∆ *E* = 7.24 ± 2.71) ( *p* ≤ 0.001, df = 2, 27, *F* = 13.815), while the two polishing groups were not statistically significantly different ( Fig. 4 ).

**Fig. 5:**
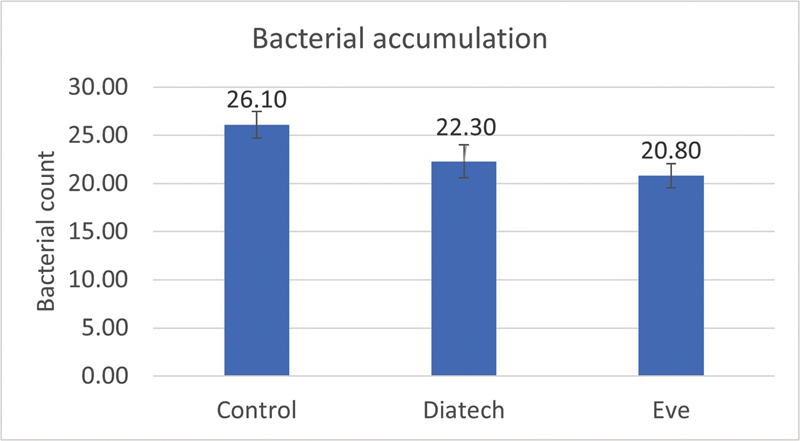
Bar chart representing mean values of bacterial count.

For the bacterial count, the control group (26.1 ± 1.91 CFU) was statistically significantly different from both polishing groups (SE: 20.8 ± 1.75 CFU) and (SD: 22.3 ± 2.41 CFU) ( *p* ≤ 0.001, df = 2, 27, *F* = 17.896), while the two polishing groups were not statistically significantly different ( Fig. 5 ).

A positive correlation between color change and surface roughness was observed, *r* = 0.64 ( *p* < 0.001), and also between the color change and the surface roughness, *r* = 0.696 ( *p* < 0.001).

**Fig. 6:**
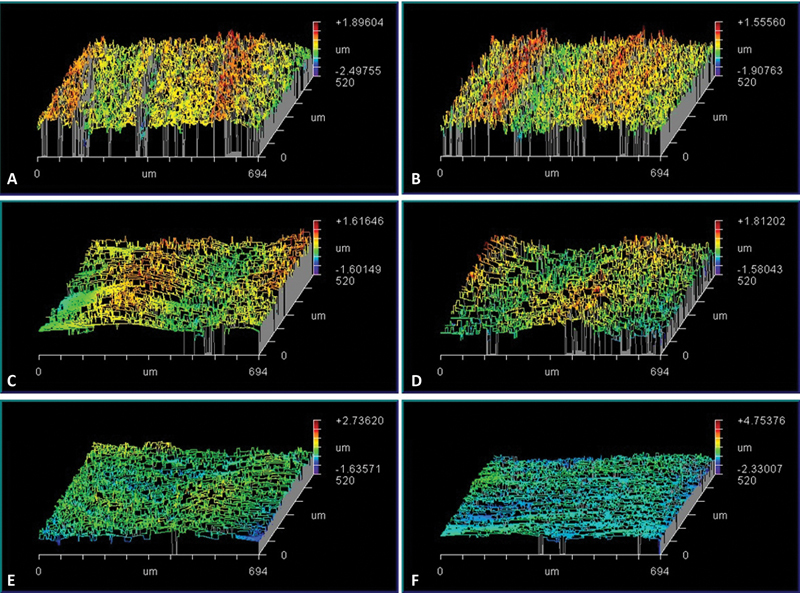
White light interferometry images ( **A** and **B** ) Control group, scaling only, ( **C** and **D** ) Scaling followed by polishing using Eve Diapro lithium disilicate polishers, and (E and **F** ) Scaling followed by polishing using Diatech ShapeGuard ceramic polishing plus kit.

Light microscopy showed that ultrasonic scaling changed the topography of the ceramic surface, resulting in deep scratches. The surfaces were smoothened by intraoral ceramic polishing, which confirms the surface roughness values after using the polishing systems. All polishing surfaces presented similar images ( Fig. 6 ).

## Discussion

The present study evaluated the effect of two different intraoral polishing systems on the surface roughness, color stability, and bacterial accumulation of ZLS ceramic. Based on these results, the null hypothesis was partially rejected as there was a statistically significant difference in the surface roughness, color measurements, and bacterial count before and after polishing; however, there was no statistically significant difference between the polishing kits.

Intraoral polishing significantly decreased the values of surface roughness; the highest surface roughness values were found for the scaling group (∆Ra = 0.52 ± 0.09 µm). Both polishing systems showed a significant decrease in the mean values of Ra, which subsequently led to a significant decrease in the values of color change showing a positive correlation. This was in agreement with previous studies that showed that the use of ceramic polishing kits was effective in reducing surface roughness. 27 28

Previous studies showed that both type of ceramic material and polishing systems affect the material's surface roughness. 29 30 31 32 According to Bollen et al, the surface roughness value (Ra = 0.2 µm) is the threshold below which bacterial adherence would not be expected. 33 However, even though the measurements of surface roughness after polishing in the present study were within the clinically acceptable range, there was no statistically significant difference in the bacterial count between the scaling and polishing groups. This may be attributed to the crystal content and the surface characteristics of the ceramic material which causes an uneven surface of the polished material after the removal of the glazed surface. The variations in the ceramic microstructures made it difficult to determine the appropriate polishing method for each ceramic. Our ﬁndings were in accordance with Limpuangthip et al 34 who found that multipurpose polishing kits reduced surface roughness of CAD/CAM ceramic materials to the similar level of the laboratory as-received samples.

In this *in* *vitro* study, only one type of ceramic was evaluated. Also, the polishing time was fixed for all groups. Different ceramic systems would probably show different results with different polishing time and techniques. To assess the surface roughness and its effect on color change, and bacterial count of various ceramic materials after polishing using various polishing products and procedures, additional research is required.

Possible confounding variables that could affect surface roughness such as ceramic material and shade, applied pressure, scaling, and polishing devices were controlled in this study. However, a limitation of this study is the use of one type of restorative material which limited the generalizability of the ﬁnding. Moreover, the oral environment may have additional effect on the surface roughness. Therefore, further studies should explore other types of CAD/CAM ceramic materials and other properties such as wear of the material and its opposing tooth to comprehensively evaluate the clinical performance of the material.
